# Wicked Labels, and the Power of Narrative in Mental Health Nursing

**DOI:** 10.1111/jpm.70040

**Published:** 2025-10-10

**Authors:** Michael Haslam, Karen Wright, Gary Lamph, Mick McKeown

**Affiliations:** ^1^ School of Nursing and Midwifery University of Lancashire UK; ^2^ School of Nursing and Midwifery Keele University UK

## 1 Introduction

As the musical and film, *Wicked* (based upon the novel by Maguire 1995), takes us firmly by the hand, and once more leads us down the yellow brick road, its cultural resonance has become irrefutable. The film in particular has drawn online vitriol from alt‐right media, prompting accusations that it is ‘woke’ (Maltby 2024) and that it promotes an ‘LGBTQ agenda’ (Williams 2024). Undoubtedly, at the heart of the cacophony surrounding *Wicked* are the film's central themes of identity and marginalisation, particularly at those intersections of race, gender and sexual orientation; the politics of which no doubt contribute to experiences of exclusion.

Amongst the many positive film reviews, Johnson (2025, np) remarks upon how *Wicked* speaks ‘directly to viewers’ personal experiences of exclusion, self‐affirmation, and activism’ in response to authoritarianism. Indeed, our own observations here are that the film *Wicked*, especially when juxtaposed with the *Wizard of Oz*, also offers a powerful allegory for the ‘othering’ of those experiencing mental ill‐health and distress. It is impossible, for instance, to overlook the film's overt examination of belonging and the construction of difference against an oppressive backdrop of patriarchal and paternalistic power structures; themes that are also of direct relevance to contemporary mental health services and nursing care.

Our paper draws upon social constructionism and *Wicked* as an allegory to consider how diagnostic labels are less objective, pre‐existing entities, as they are narratives arising from social, political and cultural processes. When considered within the context of mental ill‐health and distress, we argue here that the social construction of ‘wickedness’ in *Wicked* mirrors dominant psychiatric discourse, processes of psychiatrisation and their harmful effects. Further, despite the two films' contrasting responses to systemic oppression^1^ we suggest that when viewed together, they reinforce both the importance of *seeing beyond* damaging and pejorative diagnostic labels and the restorative role of narrative within this context.

For the sake of clarity, while our title uses the term ‘wicked’ in a direct reference to the musical and film, we also acknowledge that the intractable themes of labelling, marginalisation and othering explored in our paper are also illustrative of those ‘wicked problems’ posed by Rittel and Webber (1973). It is not our intention, however, to directly engage with this theoretical framework here. Instead, our paper will consider related notions of abjection, biopower and testimonial injustice in respect of narrative and damaging psychiatric discourse. Our own position here is not one of antipsychiatry, per se. Rather, it is in pulling back the curtain and explicating these crucial themes where we hope to both make sense of them and consider their pertinence to contemporary mental health nursing practice.

## 2 Something Has Changed: Understanding the Power of Wicked Labels

We begin this paper with the claim that the Wicked Witch of the West represents a physical embodiment of the marginalised ‘other’. While in the original *Wizard of Oz* film, the Wicked Witch is a feared and maligned figure, labelled as dangerous and unpredictable, the film, *Wicked*, subverts this portrayal, viewing the witch in a more sympathetic light. In a similar vein to other villain‐revisionist backstories, she is instead portrayed as a product of an intolerant society that has misunderstood and ostracised her rather than a symbol of inherent evil. Indeed, through *Wicked*, we are introduced to a more humane representation of the witch. She is granted a name, ‘Elphaba’, which immediately humanises her, and through the film, we become witnesses to her vulnerabilities and experiences of trauma.

Born of an extramarital affair, shunned by family members and peers alike due to her unusual skin colour, while also being blamed for the death of her mother and disability of her sister, *Wicked's* Elphaba is positioned as a tragic victim of circumstance who was ostracised from an early age. Yet, despite her own marginalisation, she is initially sympathetic towards those groups who are similarly oppressed and excluded at the hands of the great and powerful Oz, even travelling to the Emerald City to advocate for their liberation.

Meanwhile, in both films, the Wizard masquerades as a benevolent ruler, all while manipulating others using spectacle and propaganda to maintain his power. The Wizard's actions here are those of the ‘moral entrepreneur’ (Becker 1963), who, driven by personal and political interests, identifies certain groups as deviant. Seeing through this façade, however, and refusing to comply with his plans, Elphaba is immediately and very publicly denounced by the Wizard. Labelled as ‘wicked’, she is vilified herself and declared a common enemy of Oz, thus also serving political ends by becoming a focal point for societal fears. And it is in controlling this narrative and sealing Elphaba's eventual fate as the Wicked Witch of the West where the Wizard distracts from the true source of oppression, ensuring that the status quo—social cohesion and existing power structures in Oz—are maintained.

## 3 Accepting of Limits: The Social Construction and Discourse of ‘Wickedness’

In the *Wizard of Oz*, a ‘good vs. evil’ dichotomy is clearly discernible from the outset, Elphaba situated as diametrically opposed to the ‘good’ citizens of Oz and its seemingly virtuous leader. Through *Wicked*, however, we come to understand that ‘wickedness’ is less an inherent trait for Elphaba, as it is a label *thrust upon* her; a social construction, arising initially through intersectional oppression and then further perpetuated by societal fears. Way before she eventually accepts her fate as the Wicked Witch of the West, Elphaba is demonised, denied the common attributes or shared characteristics of her fellow Ozians. We suggest that this social process of ‘othering’, constructing difference as threatening, also aligns with Kristeva's (1982) concept of the abject; a notion that has proved serviceable in consideration of various aspects of psychiatric practice (Johansson and Holmes 2022, 2025; Holmes et al. 2006; Mckeown and Stowell‐Smith 2006).

Elphaba and her distinctive appearance already embodies the abject, her green skin placing her outside established, normative systems of identity, thus threatening the boundaries of societal norms and prompting public feelings of revulsion. Further, Madam Morrible's suggestion early in the film that Elphaba learns to harness her emotion, frames her burgeoning powers as dangerous forces requiring containment, rather than as legitimate responses to her distress. Nevertheless, it is in the Wizard's eventual construction of her as ‘wicked’, where Elphaba is further set apart from common humanity, given that her ‘wickedness’ is perceived to be disruptive to the symbolic order of Oz.

To the citizens of Oz, she becomes regarded as fearful and monstrous, a liminal entity that is uncontainable and ultimately incomprehensible (Shildrick 2002). Yet simultaneously, Elphaba's presence provides the comfort that *they* are *not her*, especially when confronted with knowledge of her ‘wickedness’. This process of abjection, where Elphaba is othered and demonised, mirrors the experiences of those with mental ill‐health, especially at those intersections of unhelpful diagnostic labels, emotional dysregulation and perceived risk (Wright et al. 2007; Mckeown and Stowell‐Smith 2006).

The Wizard, meanwhile, represents a figure of unquestioned patriarchal authority, although his power is later revealed to be based on performance and spectacle, rather than any real substance. Here, we suggest that there are clear parallels with bio‐psychiatry, which has historically positioned itself as the dominant arbitrator of mental health care (Kolar et al. 2023), and in doing so, has enjoyed considerable political influence and public trust. A lack of established pathophysiology and identifiable biomarkers for illness, however, as well as a poor homogeneity and temporal validity in some diagnostic labels (Kinderman 2015; Pilgrim 2001), means that bio‐psychiatry often lacks the evidence to support its scientific legitimacy (Wand 2024; Crowe 2022).

Moreover, through its own propaganda (disseminated via diagnostic manuals, professional journals and, latterly, media engagement^2^), bio‐psychiatry continues to construct and reinforce those harmful narratives, which locate the causes of mental ill‐health, mental distress and even deviance, as residing within individuals (Bracken et al. 2012). Thus, those experiencing mental ill‐health are depicted as being inherently flawed (Goffman 1961) and their behaviours pathologised, rather than their distress being seen as transient and as often arising from social determinants, or through the political and systemic misuse of power (Johnstone et al. 2018). As the psychiatric system, however, organises to project an image of itself as rationally defensible and efficacious, in doing so, it bolsters public faith that mental illness may be fixed ‘by force’ (Wand 2024, 215) and, perhaps more crucially, that *dangerous* madness can be contained.

In this way, dangerous psychiatrised individuals might also be viewed as abject phenomena, inviting a contradictory public curiosity, coupled with revulsion and fear (Mckeown and Stowell‐Smith 2006). Within this context, however, a real hazard for those experiencing mental ill‐health is the potential for the attachment of abject identities to be forever extended across wider and wider populations rather than the original extreme minority. Take for instance the ‘personality disorder’ label, which arguably functions as a clear marker of abjection, and as with Elphaba's ‘wickedness’ label, positions individuals as inherently threatening to social norms; their experiences deemed ‘unknowable’, their actions ‘unpredictable’ and testimonies often considered ‘unreliable’ (Pilgrim 2001). This echoes Kristeva's logic that phenomena or actions are identified as abject, where we lack the capability to make sense of them (Kristeva 1982).

Despite the contentiousness of some harmful psychiatric labels, however, (Porter et al. 2025; Lamph et al. 2022; Kinderman 2015; Pilgrim 2001), these reductive and apparently authoritative explanatory devices have endured, functioning to maintain social order and the established symbolic framework, thus aligning with Foucault's (1977, 1980) analyses of disciplinary and Biopower. First, the social governance role of psychiatry mandates the protection of the public and the assuaging of public anxieties surrounding risk, through the mechanisms of surveillance, control and the containment of individuals (Kolar et al. 2023).

Second, such devices not only define and maintain the boundaries of normality (Becker 1963) but also emphasise what (and who) is considered morally appropriate and acceptable. Through the categorisation and examination of those deemed ‘abnormal’ or ‘deviant’, they also operate, at least in part, as an attempt to wrest some meaning from those inexplicable behaviours, especially where the abject provokes within us an utter incomprehension. Consequently, psychiatric labels serve to defend societies against those physical, moral and psychic threats posed by firmly fixing the ‘other’ at a safe distance from the ‘self’ (Shildrick 2002), or, more pertinently, ‘ourselves’.

Hence, concurring with a long line of critics (Rose 1985; Foucault 1977; Becker 1963; Goffman 1961), we suggest that the mental health system exists with an implicit purpose of fulfilling societal and organisational agendas, prioritising the control of risk behaviours and the categorisation and resolution of those symptoms, which provoke visceral responses in the public over meeting the actual needs of those experiencing mental distress. Ultimately, in doing so, however, they reinforce those harmful ‘Normal versus Abnormal’ and ‘Them versus Us’ dichotomies (Haslam and Harding 2024), and such practices risk further generation of iatrogenic harms through the invalidation and re‐traumatisation of those in need of help and support (Beale 2022). Thus, prompting our critical reflection here of where it is that ‘wickedness’ *actually* resides.

Moreover, it is mental health nursing's overt collusion with this system, meanwhile, carrying out the ‘dirty work’ of psychiatry (McKeown 2024; Warrender 2021; Godin 2000; Emerson and Pollner 1976), that arguably positions us primarily as agents of social control. As mental health nurses, our role is one that traditionally contributes to the maintenance of the status quo, complicitly enacting institutional power under a rhetoric of benevolence, although one that masks a reality of coercion and control. The potential for engaging in this sort of work therefore forces us to betray our core values as caregivers, thus contributing to our own experiences of workforce alienation (McKeown 2024) and of moral distress (Repenshek 2009; Jameton 1984).

## 4 Someone Else's Game?: Whose Narrative Is It Anyway?

Applied to mental health nursing, Elphaba's story prompts us to scrutinise psychiatric discourse, highlighting how for some, diagnostic labels often serve little purpose beyond the maintenance of those systems of oppression that create and enforce said labels. Echoing the historic demonisation of non‐conforming women in the 17th‐century witch trials, these wicked labels can (and often do) further perpetuate the structural marginalisation of vulnerable groups, especially at those intersections of race, gender and sexual orientation (Dhari et al. 2024). Yet, it is through understanding Elphaba's eventual hostility as the Wicked Witch of the West as being an understandable response to her marginalisation, where we might similarly consider how the responses of those experiencing acute distress, and behaviours, which are deemed ‘challenging’ or ‘undesirable’ by society, are less linked to inherent flaws in biochemistry or personality as bio‐psychiatry would have us believe.

Behaviours that do not conform to social norms are likely contextual (Johnstone et al. 2018; Moncrieff et al. 2024), representing instead those natural responses to broader socio‐political and interpersonal challenges, or perhaps (mal)adaptive strategies developed by individuals to manage intense distress against backdrops of trauma and adversity (Wand 2024). Professionals are therefore likely more efficacious if responding to an individual's identified difficulties and focussing upon the contexts within which these arise, rather than simply their diagnostic label (Kinderman 2015).

Elphaba's experiences, however, also highlight the common issue of epistemic injustice in those experiencing mental ill‐health, prompting reflection around whose knowledge and perspectives are excluded from our understanding of illness. In particular, testimonial injustice, as initially coined by Fricker (2007) and as applied to those experiencing mental ill‐health (Watts 2024; Fisher 2023), occurs where subjective patient experience and stories are said to hold less value than professional judgement and objective ‘hard’ evidence, such as mental state examinations. This issue stems in part from bio‐psychiatry's assertions concerning the nature of valid knowledge, giving stronger credence to that which is objectively observed, measured and verified (Dhari et al. 2024).

Testimonial injustice is especially compounded where individuals are subjected to a ‘credibility deficit’ (contrasted with those ‘credibility excesses’ or epistemic privileges granted to psychiatry and the wider psy‐professions, Fricker 2007). A reduced degree of credibility results from stigmatising attitudes or prejudice from the hearer, further increasing the marginalisation of those already subjected to structural inequalities. Just as Elphaba's lived experience, for instance, is disregarded due to her ‘wicked’ label, so too, the stigma of harmful diagnostic labels can often lead to a systemic undermining of the speaker's authority (Watts 2024), leading to the testimonies of those carrying such labels to be subsequently doubted or misconstrued (Ware et al. 2022).

Again, considering the ‘personality disorder’ label, already critiqued for misogyny in its application (Shaw and Proctor 2005), it likewise provokes powerful value‐laden judgements, thus risking a dismissal of personal accounts. A form of moral discrimination is evident here, both ubiquitous and enduring, reinforcing a further dichotomy between those considered *not fully responsible* for their behaviours, therefore deemed deserving of care, and those considered *irresponsible*, deemed less deserving (Johnston 2010; Crowe 2008; Wright et al. 2007).

Still, when considering the problematic issue of testimonial injustice, the importance of narrative is once again highlighted, this time in its potential for remedial action. First, narrative‐led approaches encourage us to see beyond wicked labels and promote empathy. They facilitate the shift from dominant discourse concerning pathology and those reductionist, medicalised approaches sanitising individual experience, towards more contextual, meaning‐focussed approaches to comprehending distress and its origins (Johnstone et al. 2018). Indeed, films depicting the human condition within popular culture such as *Wicked* invite consideration of those broader *storied* and *storying* aspects of our being (Kirkpatrick 2008), and through the medium of story themselves, reinforce the importance of understanding personal history and the social context of behaviour (Hall and Powell 2011; Orbuch 1997). In essence, we are encouraged to attend to those narrative accounts that are traditionally excluded from our understanding.

Second, where systems that perpetuate wicked labels derive benefit from limiting individual sense of agency, it is narrative‐led approaches, meanwhile, that empower the individual through the reclaiming and re‐authoring of stories told about themselves and their own lived experiences (Orbuch 1997; White and Epston 1990). The very act of storytelling itself is intensely relational (Fisher and Lees 2015; Kirkpatrick 2008), providing the conditions for intersubjectivity through a stimulation of personal reflection and shared narratives. *Wicked* (through its juxtaposition with the *Wizard of Oz*) reminds us that it is through a re‐authoring of these individual narratives where mental health nurses and users can finally resist the systemic nature of othering, holding space for shared humanity and ultimately providing a potential antidote to fear, division and abjection (Wright et al. 2007).

## 5 Working in Tandem: A Case for Dialogic Approaches

It is also through curiosity and shared narratives where we argue that the potential for partnership working with those experiencing mental ill‐health becomes possible, offering a path towards mutual understanding and solidarity. The failed allyship, however, between Elphaba and Glinda (the good witch of the North in the *Wizard of Oz* film), is an example in *Wicked* of how this potential is not always fully realised, and further reinforces *Wicked*'s parallels with more traditional practices of mental health nursing. Despite their shared history and initial bond, Glinda still knowingly and complicitly aligns with the dominant power structures of Oz, even dismissing Elphaba's intentions to challenge the system as ‘delusions of grandeur’. She ultimately colludes with the established hierarchy, choosing ambition and societal approval over solidarity with her friend.

Despite this, the situation is not entirely without hope. The precarious nature of allyship observed through Elphaba's relationship with Glinda is contrasted with the potential for true solidarity that is later observed in the character of Dorothy in the *Wizard of Oz*. Dorothy's character symbolises compassion, enablement and liberation, a very literal embodiment of the Good Samaritan parable, physically crossing the road to help those less fortunate, and thus positioning her as the direct antithesis to the traditionally hostile and domineering Wicked Witch. It is in Dorothy's tendency to ally with those who are oppressed, however, that leads us to question, in another timeline, could this story have been very different? Could Dorothy and the Wicked Witch of the West have become Allies?

Indeed, we suggest that Dorothy has the capacity to see beyond Elphaba's ‘Wickedness’ label and to understand her motivations, while Elphaba's own backstory in *Wicked* exposes her initial sympathies for those who are similarly marginalised and oppressed. Consequently, despite their differing lifeworlds, it is not too difficult to see how under different circumstances, a potential partnership and even the possibility of allyship might have emerged. That is, if both Dorothy and Elphaba could ever overcome their initial hostilities toward each other. Extending this metaphor further to mental health nursing care, this concept resonates with the principles of relational working, where we argue that it is *also* possible to see how similar partnerships might emerge. That is of course, if those delivering care are finally able to liberate themselves from those systemic constraints that often favour and prioritise coercion and paternalistic control over the lived experience of individuals.

This, however, requires a deliberate effort to navigate those tensions inherent to our role, particularly the balancing of legal and professional duties to manage risk with the ethical imperative to uphold patient autonomy. As a divergence from paternalism and recourse to othering, we therefore suggest that attention to both radical empathy (Givens 2021) and open dialogue‐type approaches (Seikkula et al. 2006) align with this vision, providing not a single solution but a continuous effort to prioritising understanding and individual preferences within the constraints of the current system. Respectively, their ethos challenges dominant narratives perpetuated by bio‐psychiatry, which currently construct some individuals as ‘less than’. Both consistent with narrative frameworks, they advocate instead for those approaches that are more situated, dialogic and which are grounded in personal agency and subjectivity. Beyond merely seeking to categorise, reduce and control those behaviours arising from distress, for instance, mental health nurses should perhaps instead be encouraged to simply bear witness to them, sit with the discomfort and merely seek to understand rather than to resolve (Wright et al. 2007).

Acknowledging, however, that this necessitates a recognition of the sustained emotional toll such work can take on our profession (Randall and McKeown 2013; Hochschild 1983), especially where this work is further compounded by existing resource limitations, harmful workforce deficits and systemic constraints. Adequate support systems are crucial, therefore, to mitigating the risk of burnout, compassion fatigue and moral injury (Beale 2022; Edward et al. 2017) and to ensure that mental health nurses are better equipped to engage in this emotionally demanding work. Truly democratised working with those who are psychiatrised and labelled by the system would nevertheless shift the focus from coercive treatments towards those more relational and co‐produced approaches to care that acknowledge power asymmetries, validate the lived experience of those experiencing mental ill‐health and ultimately set us on our own road to challenging those systemic causes of epistemic and social injustice.

## 6 A Chance to Fly: A Call for Solidarity and Collective Emancipation

Reclaiming narratives is a first act of resistance, going some way to supporting the contestation of those existing patriarchal, racist and other societal power structures preserved via bio‐psychiatry. A call for emancipation by a growing and very vocal survivor movement already highlights the urgency of this (Wand 2024). Despite their diversity in experiences, survivors and refusers of psychiatric and mental health services are united both in the rejection and the challenging of those harmful narratives and practices (Bracken et al. 2012; Shaw and Proctor 2005) that have systematically contributed to their own experiences of othering and of social and epistemic injustice.

Despite some real differences in positioning and experiences between nurses and survivors, users and refusers of services, we further suggest that genuine solidarity and allyship are possible, especially given that we are, after all, inextricably a part of each other's narratives (Haslam 2024; Kirkpatrick 2008). And it is in this paradoxical space where a mutual commitment to collectively resist harmful orthodoxies and challenge constructions of ‘otherness’ (Wright et al. 2007) can emerge and be nurtured. Such potential certainly has the capability to disrupt those psychiatric discourses that pathologise natural human responses to adversity. Ultimately, we suggest that this move would also address the cognitive dissonance inherent in our role, encouraging for nurses a renewed sense of meaning and purpose and directly reducing alienation and moral distress among the workforce.

## 7 Making the Leap and Defying Gravity: Concluding Thoughts

Concluding this paper, we contend that *Wicked* is much more than a reimagined fantasy tale. Rather, it might be viewed as a socio‐political call to action (Johnson 2025), through which we are encouraged to actively interrogate the foundations of uncontested authority, and those existing power structures that are sustained through paternalism and the marginalisation of others. Applied to mental health nursing, and especially where the dehumanising effects of labelling are highlighted, we encourage nurses to eschew existing dichotomies, recognising that these are founded upon unhelpful diagnostic labels and are perpetuated by the pathologisation and medicalisation of distress. Central to this is an appreciation of ourselves as inherently *storied* and *storying* beings, and the reclamation and re‐authoring of personal narratives that support the recognition of shared humanity across clinical boundaries, while challenging social constructions of ‘otherness’.

Just as Elphaba's anthem, *Defying Gravity*, is a pivotal moment in her own story and signifies a reclamation of her own personal narrative and identity regardless of the consequence, her experience can be seen as echoing those of mental health survivors and users of services. This individual act of defiance and narrative ownership, however, might also represent a turning point in our own stories as mental health nurses. The lyric, ‘together we're unlimited’, especially reminds us that going beyond simply understanding—that it is in both aligning and allying ourselves with users of services and survivor groups, where mental health nursing might also find its strength and a renewed sense of purpose. Should we choose to do so, we are presented with a mutual opportunity to finally take the leap, disrupt reductive psychiatric discourse, and together become a future force for systemic change.

## Ethics Statement

The authors have nothing to report.

## Conflicts of Interest

Mick McKeown is an editor of the *Journal of Psychiatric and Mental Health Nursing* and a co‐author of this article.

## Data Availability

Data sharing not applicable to this article as no datasets were generated or analysed during the current study.

## Endnotes

^1^
Where the *Wizard of Oz*'s ‘Over the Rainbow’, for instance, represents a passive yearning for a different world free from oppression, it is *Wicked*'s ‘Defying gravity’ that carries a sense of urgency, challenging us to rise up in an act of resistance and to liberate ourselves from existing oppressive narratives.

^2^
Consider the wide‐ranging scholarly and social media engagement with the work of critical psychiatrist Joanna Moncrieff, particularly her debunking of the ‘chemical imbalance’ hypothesis of depression and the backlash from psychiatrists and pharmaceutical industry (Lane 2025).
